# Developing Virtual Reality Trauma Training Experiences Using 360-Degree Video: Tutorial

**DOI:** 10.2196/22420

**Published:** 2020-12-16

**Authors:** Devika Patel, Jessica Hawkins, Lara Zena Chehab, Patrick Martin-Tuite, Joshua Feler, Amy Tan, Benjamin S Alpers, Sophia Pink, Jerome Wang, Jonathan Freise, Phillip Kim, Christopher Peabody, John Bowditch, Eric R Williams, Amanda Sammann

**Affiliations:** 1 Department of Surgery University of California, San Francisco San Francisco, CA United States; 2 School of Medicine Stanford University Stanford, CA United States; 3 School of Medicine University of California, San Francisco San Francisco, CA United States; 4 Department of Neurosurgery Brown University Providence, RI United States; 5 School of Medicine University of California, Davis Sacramento, CA United States; 6 School of Engineering Stanford University Stanford, CA United States; 7 School of Public Health University of California, Berkeley Berkeley, CA United States; 8 Department of Emergency Medicine University of California, San Francisco San Francisco, CA United States; 9 J. Warren McClure School of Emerging Communication Technologies Scripps College of Communication Ohio University Athens, OH United States

**Keywords:** virtual reality, cineVR, 360-degree video, trauma training, medical education

## Abstract

Historically, medical trainees were educated in the hospital on real patients. Over the last decade, there has been a shift to practicing skills through simulations with mannequins or patient actors. Virtual reality (VR), and in particular, the use of 360-degree video and audio (cineVR), is the next-generation advancement in medical simulation that has novel applications to augment clinical skill practice, empathy building, and team training. In this paper, we describe methods to design and develop a cineVR medical education curriculum for trauma care training using real patient care scenarios at an urban, safety-net hospital and Level 1 trauma center. The purpose of this publication is to detail the process of finding a cineVR production partner; choosing the camera perspectives; maintaining patient, provider, and staff privacy; ensuring data security; executing the cineVR production process; and building the curriculum.

## Introduction

### Virtual Reality in Medical Education

Emergency and inpatient medical training has historically occurred in an apprentice style of teaching. The famous adage “see one, do one, teach one” describes the process where trainees would observe a procedure once, perform that procedure themselves, and subsequently teach others how to perform it. However, this learning process traditionally takes place in the context of real patient care, which inevitably results in increased medical errors as the learner practices their skills [1].

Fortunately, over the last 2 decades, medical education has embraced the use of simulated environments as a way to improve the competency of trainees while decreasing medical errors. This not only increases patient safety but also improves the learner’s clinical skills by allowing for repetitive practice of the intended skills coupled with specific and informative feedback that results in better skills performance [1-3].

Simulation-based medical education uses actors, mannequins, or trainers to create an artificial representation of a real-world process to facilitate experiential learning [4]. Simulation has been shown to improve teamwork and communication across high-risk industries and a variety of medical disciplines including anesthesiology, surgery, obstetrics, emergency medicine, pediatrics, and critical care [5,6]. Although medical simulation is an effective and well-established teaching tool, most methods depend on expensive models, require specific equipment and manpower, and are commonly considered to be low-fidelity and artificial to allow suspension of disbelief and immersion in the simulation [6-10].

Virtual reality (VR)–based medical education offers a potential solution to these traditional simulation limitations. VR allows learners to immerse themselves in an authentic and accessible clinical scenario using 360-degree video VR and audio technology (cineVR), without compromising patient safety [11,12]. CineVR is a subset of VR that depicts a real (versus computer-rendered) environment where the viewer can look around the room and see in all directions but cannot interact directly with the scene. Unlike traditional simulation-based education, cineVR allows for flexibility, independent learning, and unlimited number of opportunities to practice and hone teamwork, communication, and patient management skills. While in cineVR, the learner is not interacting with a virtual environment, but with the authentic, real-world environment of the 360-degree video. CineVR allows learners to gain familiarity with the environment before being there in real life, and a qualitative study has shown that the realism associated with cineVR can elicit emotional responses [13].

A growing number of academic medical centers are using VR to augment their training curricula [14,15]. VR technology is being used widely in surgical education. Several randomized controlled trials have found that practicing surgical skills in VR improved technical performance in the operating room [16,17]. CineVR has been used in a variety of settings, including naloxone administration training, where it was shown to improve knowledge and perceptions about opioids and use of naloxone, and in diabetes education, where it was shown to improve cultural self-efficacy and diabetes attitudes among health care providers and administrators [13,18]. These technologies are also being applied with companies such as Embodied Labs, exploring educational topics such as patient communication. An early study suggests that participants that watched one of the Embodied Labs simulations had positive learning experiences, demonstrated empathy, and reported an increase in their perceptions of using 360-degree VR as a learning tool [19].

### Virtual Reality in Trauma Care

Medical and surgical trainees have limited opportunities to safely practice trauma response skills without compromising patient safety. Trauma care is defined as care for critically injured patients, where patient care scenarios can include major traumatic injuries such as motor vehicle collisions or gunshot wounds [20]. High-level trauma resuscitations require coordination across multidisciplinary care teams in an unpredictable and chaotic environment. For most medical trainees, trauma care training occurs on the job, with the exception of a 2-day Advanced Trauma Life Support (ATLS) training course that is renewed every 2 years and includes 1 day of simulation training with standard actors. The ATLS course focuses on the process and necessary procedural skills to evaluate and care for an injured trauma patient. While surgical residency programs sometimes implement simulation-based trauma care practice, these are limited by cost and duty-hour regulations and not guided by standardized best practices or strategies [21]. The majority of trauma instruction is therefore variable, with trainees learning through experiential learning as an observer or participant in live traumas.

This method of trauma training is suboptimal for 3 reasons. First, it leads to a cohort of trainees with variable experience and facility in managing trauma patients [21]. Second, it puts learners in a highly stimulating and stressful environment without experience or stress management techniques, which can decrease clinical decision-making capacity [22,23]. Third, learners often do not receive adequate training in leading, managing, or coordinating the other medical providers in the room [24].

### Project Goal

The goal of this project was to develop a trauma training curriculum, to be used as standalone or part of standard ATLS training, for residents by using 360-degree videos of real trauma care scenarios, to address the limitations of traditional trauma care training. The curriculum focuses on skills such as teamwork, the management of a trauma patient, understanding and empathizing with the roles and priorities of different team members, and anticipating next steps in care planning. This article specifically discusses the process and challenges related to (1) finding a production partner, (2) selecting recording perspectives, (3) equipment selection, (4) setting up and filming the 360-degree videos, (5) file management and video editing, (6) data protection and privacy, and (7) curriculum development.

In this article, we describe our process for planning, filming, and producing trauma care training videos. We hope that, in doing so, we demonstrate the feasibility for a small group to explore VR simulation and encourage other groups to undertake more VR filming projects. To that end, we highlight both challenges and lessons learned, as well as our recommendations for future work, so that this paper might be used as an open reference tool for future projects.

## Methods

We filmed 20 live and simulated high-level trauma resuscitations in one trauma bay at the Zuckerberg San Francisco General hospital over 6 months. This project was a collaboration between the departments of trauma surgery, anesthesia, emergency medicine, nursing, and hospital facilities, and filming and editing were performed by colleagues at the Game Research and Immersive Design (GRID) Lab at Ohio University. Funding for this effort was obtained through the San Francisco General Hospital Foundation. Human subjects protection was granted through the Institutional Review Board (IRB) of University of California, San Francisco. The resuscitations were filmed from 6 different perspectives to capture the experiences of 6 different types of providers. The footage from 8 of these trauma scenarios was used to produce cineVR videos, 4 of which were further edited to include a trauma curriculum for trainees. In this section, we describe our methodology for making these videos, from planning to execution.

### Choosing a Production Partner

Most health systems do not have the experience or skills to record, edit, and produce 360-degree video and audio simulations. Therefore, video production necessitated collaboration with an outside partner. This required additional funding, which our team was able to procure through grants.

Due to the highly sensitive nature of storing real patient scenarios, a significant challenge required identifying a partner institution with a Health Insurance Portability and Accountability Act (HIPAA)–compliant data security system. To find a partner, we conducted an online search, asked experts in the VR field, and reached out to both research centers and production companies to understand their capabilities, data security infrastructure, cost, health care experience, and timeline. A search of local companies revealed that commercial VR production entities rarely had HIPAA-compliant data editing and storage systems. After an extensive search, we identified the GRID Lab at Ohio University. The GRID Lab, created in 2005, focuses on the research and development of Virtual, Augmented, and Mixed reality experiences; serious and educational games; simulations; computer animation; and motion capture [25]. As an academic institution, they possessed extensive experience in working in the health care space developing cineVR technology. Thus, their institution had standardized infrastructure for being HIPAA-compliant.

### Selecting the Recording Perspective

Our project aimed to increase teamwork and empathy by allowing learners to experience the perspectives of their colleagues through cineVR. Therefore, an essential component to early stage planning was selecting the viewer perspectives that might allow for this experience. To allow viewers to experience their colleagues’ perspective, we identified the key personnel to capture in the cineVR as (1) team “leader” (usually a trauma surgery resident or emergency medicine resident), (2) nurse, (3) airway provider (usually an anesthesia resident), (4) attending physician, (5) charting nurse, and (6) patient. We intended to capture the traumas from each of these perspectives, generating 6 videos for every trauma event.

**Figure 1:**
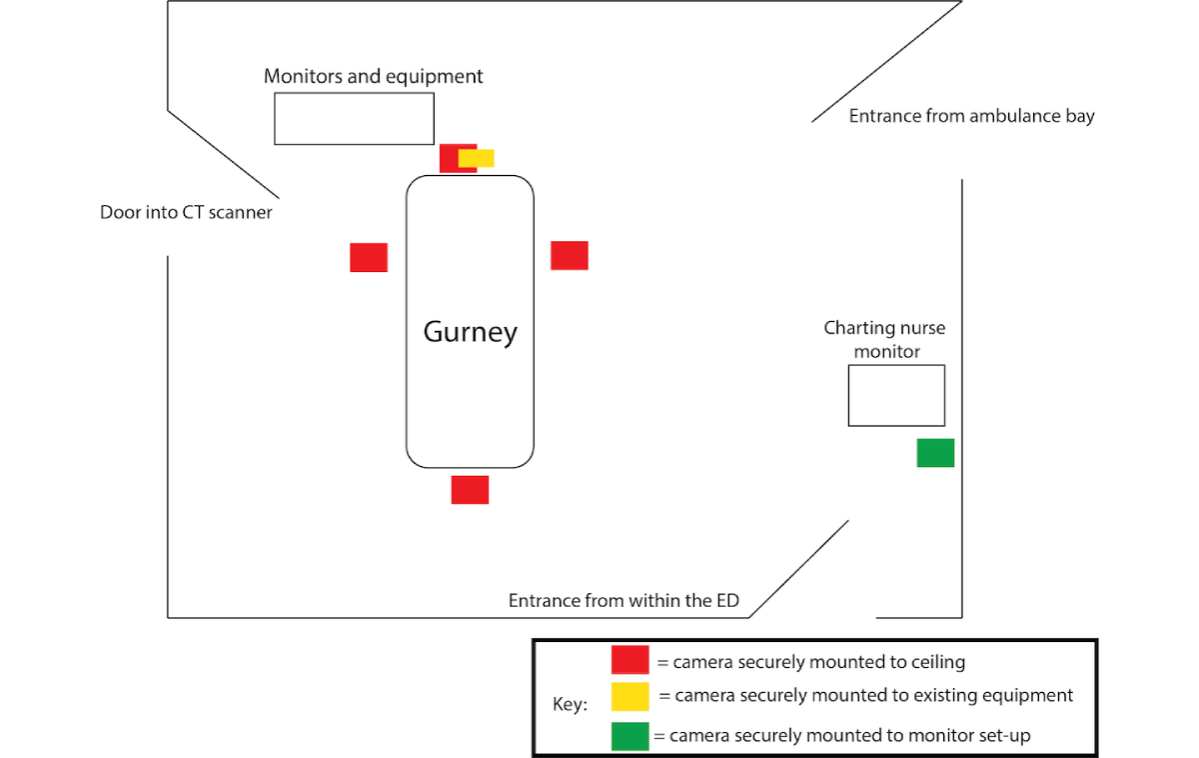
Schematic of equipment set-up in the selected trauma resuscitation room. CT: computed tomography; ED: emergency department.

In the general practice of trauma care, specific providers often stand at certain sides of the patient. For instance, airway management will primarily be at the head of the bed. Therefore, we chose our camera locations based on the providers we had identified and mounted cameras not only to the left of the bed and the head of the bed, but the right of bed (nurse perspective), foot of bed (attending physician perspective), periphery of the room (charting nurse perspective), and the patient gurney (patient perspective; Figure 1).

### Equipment Selection Considerations

There are many commercially available cameras with the ability to film in 360 degrees. We considered the following when evaluating potential equipment.

#### Image Quality

Quality varies greatly between cameras and at different price points. To make our videos as immersive as possible, we chose cameras that record video in 4k (a resolution of 3840 x 2160 pixels, compared to standard HD video of 1920 x 1080 pixels) to ensure high-fidelity output.

#### Auto-Stitching

360-degree videos are created by “stitching” together 2 or more separate video files. Manually stitching these files together is resource-intensive and time-intensive; therefore, we chose a camera with an “auto-stitch” feature, which allows for simple and streamlined video processing.

#### Equipment Size

Cameras are not the only equipment needed for filming; clamps, mounts, and booms are also a standard part of the unit. In an already-cramped resuscitation room, extra equipment can easily disrupt workflow. As we needed 6 equipment units in the room at once, we selected cameras that were as small as possible.

#### Ease of Use

To expedite filming, we preferred equipment that was intuitive to operate and required minimal additional training.

### Equipment Selection

After considering these 4 features, we selected Yi Technology 360 VR cameras (Yi Technology, Shanghai, China). The cameras produce high-quality images, have an “auto stitch” ability, are small and lightweight, and are easy to use. Additionally, the cameras allow for remote monitoring by streaming to a smartphone. We paired each camera to a Samsung Galaxy (Samsung C&T Corporation, Songpa-gu, Seoul, South Korea) smartphone, which functioned as a monitor. These smartphones were kept offline for data security.

We similarly selected Zoom H2N (Zoom Corporation, Chiyoda City, Tokyo, Japan) microphones because of their high-quality recording (96 kHz sample-rate/24-Bit resolution in 360 degrees), small size, and intuitive design. These microphones recorded audio in an “ambisonic,” or 360-degree pattern, as opposed to “stereo,” which records in only 2 directions. When an ambisonic audio recording is stitched to a 360-degree video, the audio and video replicate a real-world experience. The audio is able to rotate in relationship to the visual object that is creating the sound. For example, if someone on one’s right is talking in the video, one would hear a voice coming from their right-hand side.

To ensure the audio and video matched, the audio recorders were mounted in the same location as the cameras. All video and audio were downloaded to a laptop purchased specifically for this project. We chose an MSI laptop (Micro-Star International Co. Ltd., New Taipei City, Taiwan), a popular gaming laptop brand, for its high-speed performance and reliability. The MSI laptop has an Intel Core i7-10750H 2.60 Ghz processor, 8GB of RAM, internal 256 GB M.2 2280 Plce NVMe SSD storage, the GeGorce GTX 1650, and graphics card.

Additional detailed descriptions of our decision-making process on choosing the cameras and microphones are in Multimedia Appendix 1.

### Setting Up and Filming the 360-Degree Videos

We selected a single resuscitation room for filming. This section describes our considerations and approach to positioning and mounting the equipment and to testing the room set-up to ensure it did not interfere with provider workflow.

#### Room Set-Up

To set up the room, we had to consider camera positioning, ceiling hanging equipment, and mounting to existing equipment.

It was necessary to ensure that the equipment would not interfere with patient care. In selecting camera position, we primarily considered provider workflow, selecting locations that would not disrupt view of or access to the patient. However, we also considered the potential for the camera to obstruct the path of a large portable machine, such as an x-ray machine. As an additional safety measure, we planned to mount our equipment such that any camera could be rapidly pushed out of the way in the case of an unexpected emergency.

Our production partner visited several months before filming to select tentative camera positions and to prototype equipment mounting systems. We selected 2 methods to mount equipment, depending on the camera location: hanging equipment from the ceiling or mounting on existing equipment in the room. To ensure compliance with hospital policy, the director of facilities met with our team to discuss rules and weight restrictions for hanging camera equipment.

**Figure 2:**
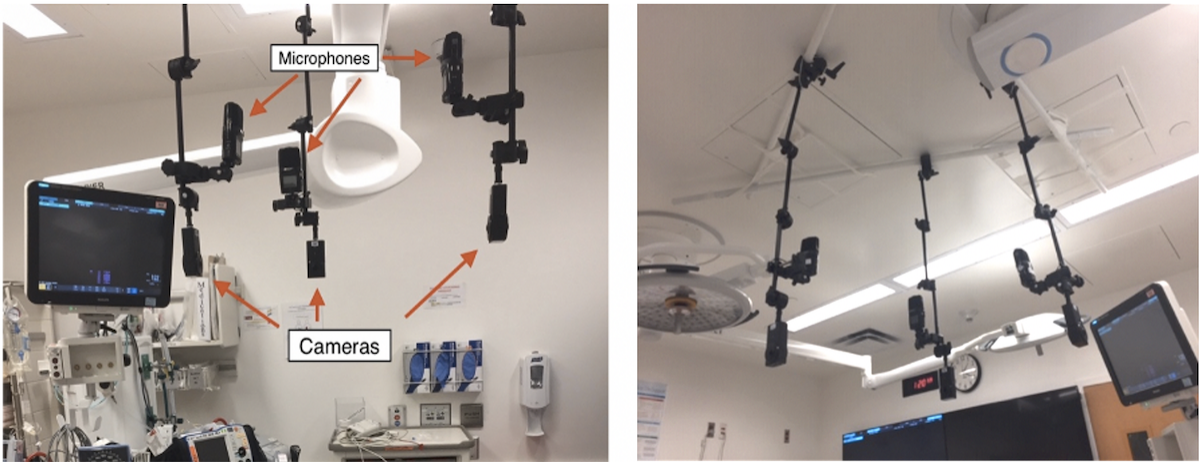
Ceiling camera configuration.

Cameras capturing the “head of bed,” “right of bed,” and “left of bed” perspectives were hung from the ceiling. We obtained approval from the hospital facilities department for our ceiling-mounted cameras. We mounted the camera using an Impact brand clamp (Gradus Group LLC, Secaucus, NJ), which was attached to boom poles and hung from ceiling hatches using wires (Figure 2). We weighed this setup to ensure it did not surpass weight restrictions. The entire ceiling mount was then covered with white gaffer’s tape in an attempt to make it less distracting to providers. All clamps and all extension arms had an additional safety tether for added security.

The additional camera and microphone units were mounted to existing equipment in the resuscitation room.

A camera was placed at the foot of the bed to capture the perspective of an attending physician standing at the foot of the bed; we mounted a camera to an overhead lamp just over the attending physician’s head. To do this, we used a clamp to attach the camera to a 4-inch long, 0.5-inch diameter metal rod; then, we used white gaffer’s tape to attach this rod to the lamp. A safety wire was added to attach the camera to the lamp arm. 

**Figure 3:**
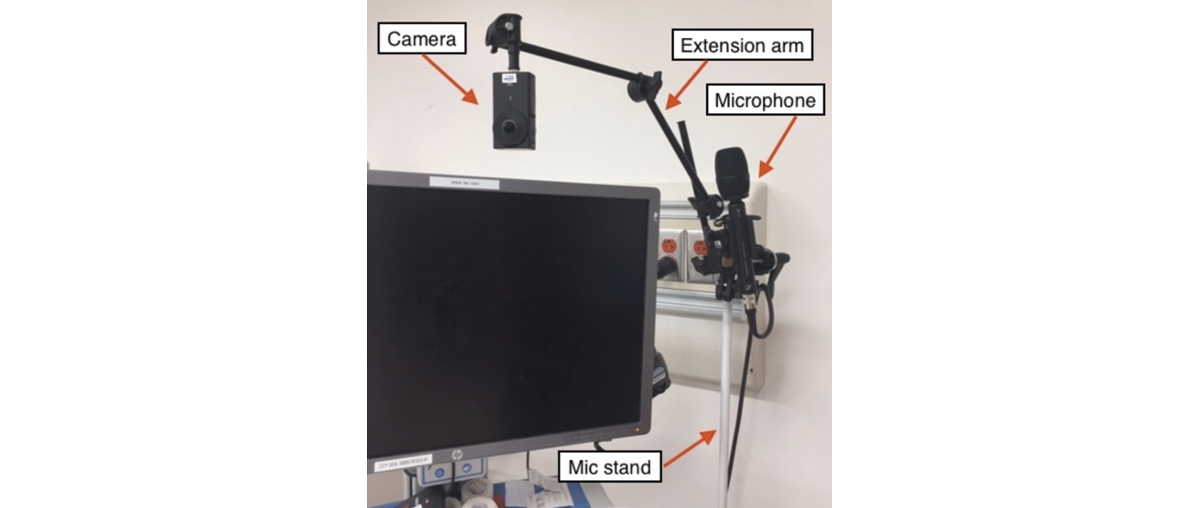
From the charting nurse perspective, the equipment configuration using a mic stand and extension arm.

To mount the camera at the charting nurse station, we used a Sennheiser AMBEO microphone unit (Sennheiser electronic GmbH & Co, Hanover, Germany) on a weighted base (7 pounds), which we placed on the floor next to the charting nurse station. We affixed the camera to an extension arm, which we then directed over the computer monitor to approximate the point of view of the nurse (Figure 3).

The camera angle from the patient gurney attempted to capture the view of the patient looking up at the trauma care team. We attached the camera and a complete Sennheiser AMBEO microphone unit to a Manfrotto camera extension arm (Manfrotto, Ramsey, NJ), which was affixed to the underside of the gurney using an Impact Super Clamp.

#### Testing the Filming Set-Up

Prior to recording, with the approval of the emergency department leadership, we closed the previously selected resuscitation room for 2 hours to run trauma simulations with emergency medicine, surgery, anesthesia, nursing, and radiology teams to obtain feedback on the camera position. This feedback was used to revise positioning of the equipment. We also reviewed sample video from each camera position to ensure it captured the appropriate perspective while remaining nonobstructive to patient care.

Two unforeseen issues that were addressed during this testing were provider height and obstruction of the airway-focused provider’s workflow.

Regarding provider height, cameras were attached between a 5’11’’ to 6’2’’ height when possible (to approximate the view from a someone of that height viewing the patient). Providers at or above this height occasionally found ceiling-mounted cameras interfered with their space. To address this, taller providers were briefed before they entered the trauma bay. Overall, most providers and staff acclimated quickly.

**Figure 4:**
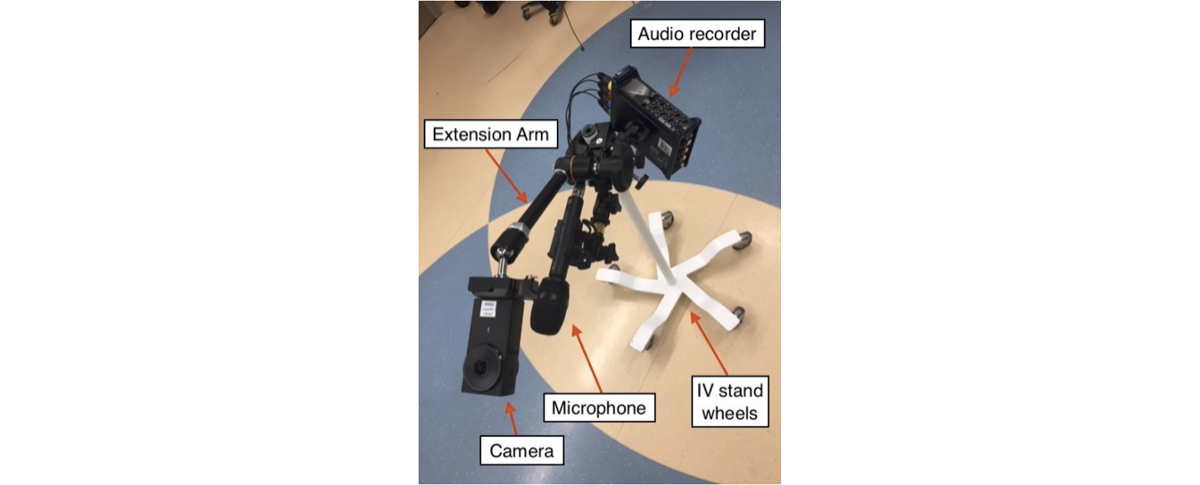
From the patient perspective, the equipment configuration using an IV stand on wheels.

Regarding airway-focused providers, capturing the anesthesiologist perspective was difficult because the provider contended both with the “head of bed” camera from the ceiling and the “patient perspective” camera attached to the gurney. To mitigate the equipment obstructing the anesthesiologist’s workflows, the production team mounted the camera and microphone unit to a wheeled IV stand, allowing the provider to push the patient perspective camera out of the way when necessary (Figure 4).

#### Filming Process

We filmed over 2 periods in a single trauma resuscitation room, first for 10 hours per day for 4 days and subsequently for 24 hours a day for 6 days. The 2 periods of filming were chosen to maximize the potential number of potential resuscitation events. Due to the unpredictable nature of trauma care, we were unable to capture the desired library of trauma cases during this initial filming period. We therefore planned a second, longer film period. During the second period, we increased filming efforts from 10 hours per day to 24 hours per day. While our goal was to capture real patient-care scenarios, we had anticipated not being able to capture our proposed set of videos in real life and recorded simulated traumas with patient actors using a real trauma team in the resuscitation room before each of the 2 live filming periods began. By filming simulations prior to capturing the live resuscitations, we were able to practice what real filming would entail, allow for the production team to have a dry run in the trauma bay to learn the workflows, and allow for any last-minute issues to be resolved. It was necessary to ensure that filming was not going to compromise real trauma care.

**Figure 5:**
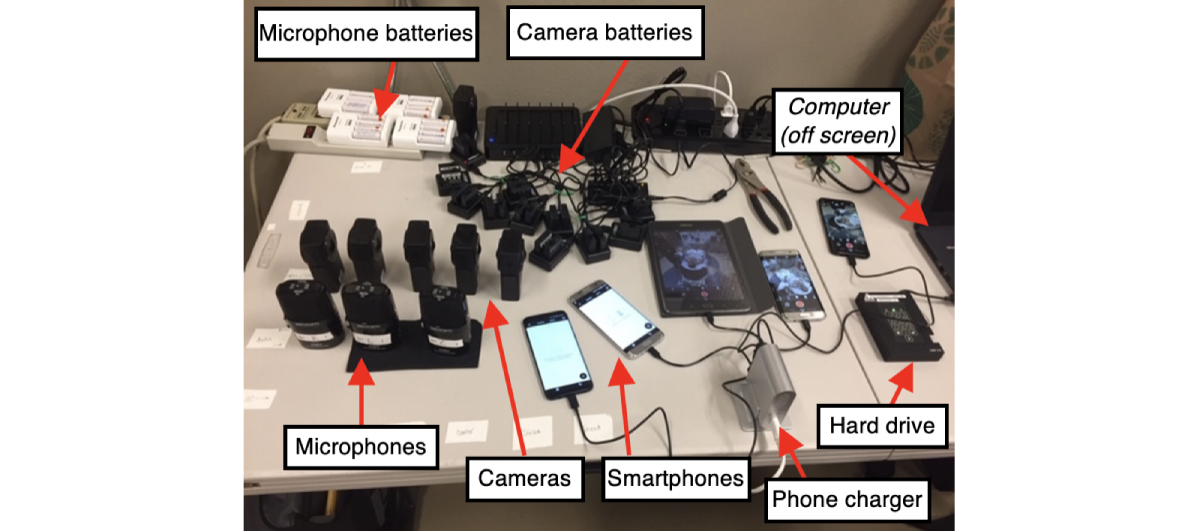
CineVR Control Station, where all equipment was placed during filming.

The team was assigned to work on production in 10- to 12-hour shifts. Each shift was composed of a 6-person team that included 3 production staff, 1 supervisor, and 2 research assistants. We created a control center near the resuscitation room where the 3 production staff monitored the equipment during recording: 2 focused on video and 1 focused audio (Figure 5). The control center also housed 1 extra set of equipment, kept fully charged and with memory cards available, 1 cell phone to monitor each camera, and a data log for all files and notes for each case. When the team was alerted of a trauma, they would enter the room and quickly turn on all 6 cameras and microphones.

#### Equipment Redundancy

After each trauma, cameras and microphones needed to be cleaned, batteries and memory cards replaced, and data uploaded to the secure server. In order to be ready for filming whenever a trauma entered the room, our team employed a strategy of equipment redundancy. Six cameras with corresponding microphones were positioned in the trauma bay, another full set was on stand-by in the control center, and 3 extra camera and microphone units were available as backup. Following each trauma event, the production team swapped out the cameras and microphones in the room to ensure new equipment was ready to use in the case of another trauma.

### File Management and Video Editing

Each filmed trauma event generated multiple files. Once videos were appropriately named and catalogued on an encrypted laptop, they were transferred to a secure, HIPAA-compliant, cloud-based storage system. In this section, we detail our file management system and review our brief video-editing process.

#### File Management

As described in earlier sections, the Yi camera automatically stitches together 2 video inputs into a single 360-degree video file. The maximum length of this video file is 4 minutes. After 4 minutes, the camera continues to record, but it generates a new file. Therefore, during an 18-minute recording, the camera creates 5 files: 4 files with 4-minute duration and 1 file with 2-minute duration. The Zoom microphone records audio as a single 360-degree audio file with no maximum length. Therefore, each camera and microphone unit recording an 18-minute trauma produced 6 total files: 5 video files and 1 audio file. For every trauma, we recorded from 6 camera and microphone positions. Hence, an 18-minute trauma would produce 36 separate files, 6 from each camera and microphone unit. To organize these files, we created a systematic labelling system, with patient number, recording device, location in room, and file sequence (A, B, C, D, etc).

#### Video Editing

Our goal was to replicate the experience of being in a real trauma room; therefore, we did not alter the video content to preserve the authenticity of the experience. We used Adobe Premiere Pro (Adobe Inc, San Jose, CA) to combine the disparate video files and any other video editing. For each video, the individual camera files were organized into the correct chronological sequence, then synchronized with the microphone audio file. Visible entryways were blurred to ensure unconsented passersby would not be included in the final product. A title card and countdown were added to the beginning of the video. The files were then transferred to Oculus Go (Oculus, Menlo Park, CA) VR headsets for testing, chosen for their portability and low cost.

The production partner and research team must align and work together to ensure that file management is well-understood and data security guidelines are followed by both teams.

### Data Protection and Privacy

It was essential to ensure patient privacy because patient care was being captured on camera to create the curriculum. IRB approval was obtained through the University of California, San Francisco. The IRB addressed the following issues: consent, data collection, storage of data, sharing of data, editing of data, and study procedures.

#### Obtaining Consent

We needed to obtain consent from everyone who would be captured on film. This included patients, providers, other hospital staff, and emergency medical services (EMS) personnel.

Developing a process to consent all providers responding to the trauma was a challenge and demanded proactive and retroactive effort by research staff. We sent emails describing the study to providers prior to their shifts. We also worked with the administrator on duty and nursing managers to organize in-person meetings during provider shifts about the filming project. During these meetings, study coordinators described the study, answered questions, and obtained signed consent. Providers and staff who did not provide consent were reassigned to a different resuscitation room for the shift when filming was occurring.

We were unable to get consent from some providers prior to filming, such as EMS personnel. To obtain retroactive consent in these cases, we stationed 1 research assistant at each of the room’s 2 entrances to identify unconsented providers and obtain informed consent.

**Figure 6:**
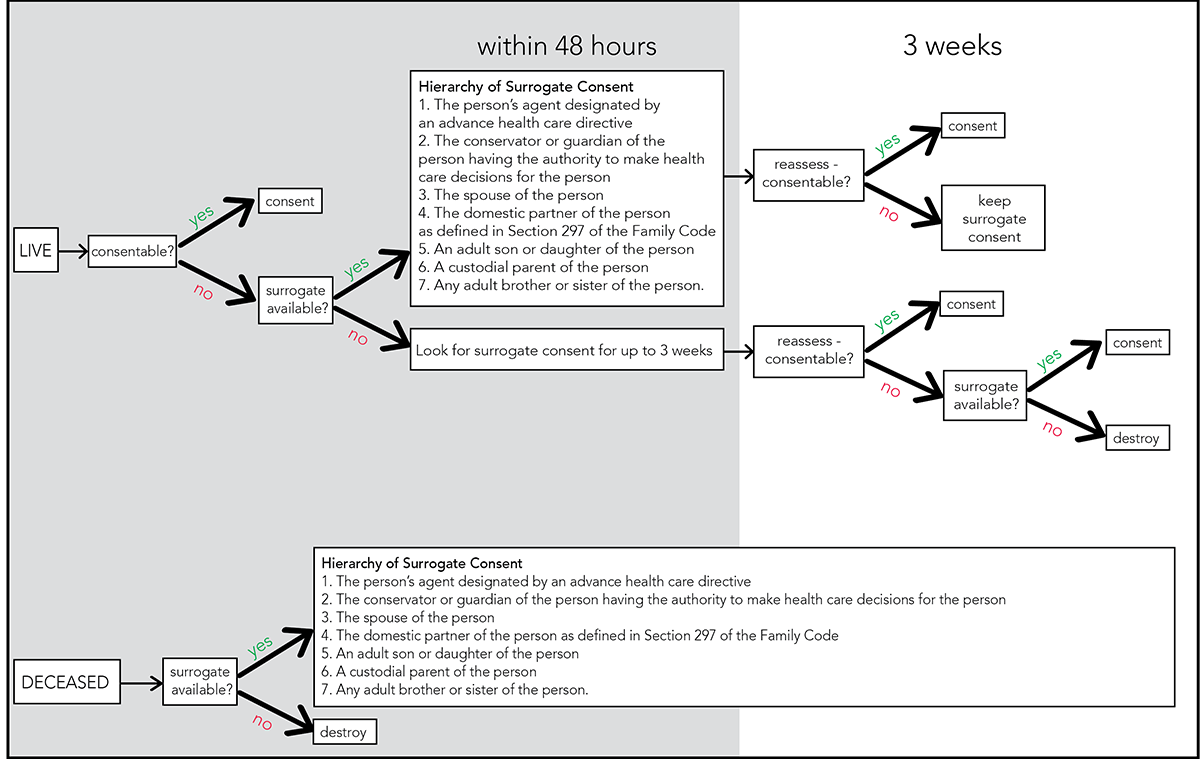
Consenting structure for patients, detailing the hierarchy of surrogate consent.

The time-sensitive nature of trauma care prevented our team from consenting the patient prior to filming. When possible, we requested consent from the patient after filming. If the patient was unable to provide consent due to severe disability, we attempted to obtain consent from a surrogate. In these instances, we referred to our institution’s hierarchy of surrogate consent. In other cases, we would reassess patient status and seek to obtain consent directly from the patient up to 3 weeks after filming, described further in Figure 6.

#### Secure Data Collection and Storage

We consulted with data and security officers in the information technology and compliance departments at our institution to develop a secure data storage and transfer process.

After each filmed trauma event, captured media files were uploaded from the devices’ SD memory cards to a laptop and backed up to a hard drive. Files were then deleted from the SD cards. At the end of each day, files were uploaded from the encrypted laptop to a HIPAA-compliant, cloud storage system. Once stored in the cloud, all files were deleted from the hardware.

Video editing was performed at the GRID Lab at Ohio University. The videos were downloaded from the cloud storage onto an encrypted hard drive, which was used to transfer the files over a secure network to a HIPAA-compliant computer in a private editing room reserved specifically for this project. The videos were edited on these secure computers, transferred back to the cloud storage system, and then deleted from all hardware.

Access to the cloud storage system was limited to 3 personnel at the GRID lab, and a maximum of 2 videos were downloaded at a time. All data repositories, including laptops, hard drives, and cloud-based storage, were encrypted in accordance with institutional protocol.

To ensure data security and privacy, work closely with your institutions’ IRB office, legal team, and data and security officers in the information technology and compliance departments of your institution to craft an IRB and appropriate partnership agreement that complies with all data security measures.

### Curriculum Development

The videos were reviewed by the research team, and 8 videos were selected to be further edited based on the visual quality and educational potential or the applicability to various trauma care providers of the video. A multidisciplinary team was assembled that included the research team, a trauma surgeon, an emergency medicine physician, an anesthesiologist, and a Vice Dean of Medical Education to develop a curriculum to overlay on the videos.

### Using Cognitive Load Theory to Inform the Curriculum

Principles of Cognitive Load Theory (CLT) were used to build the curriculum. CLT posits that learning happens best when instructional materials direct cognitive resources towards activities relevant to learning [26]. CLT describes 3 types of cognitive load: intrinsic load (essential to the learning task), extraneous load (nonessential to the learning task and often caused by poor design), and germane load (work put in to enhance schema formation). This framework was used to augment the 360-degree videos with visual and auditory overlays to train learners to focus on relevant inputs and to ignore the irrelevant inputs. Relevant and irrelevant inputs were defined through learner and expert interviews. To identify the irrelevant stimuli that contribute most significantly to the extraneous load, 28 junior residents from surgery, emergency medicine, and anesthesia were asked to view 2 trauma resuscitation videos in a VR headset. Each resident was given 4 prompts to think about and reflect on while watching the video, known as a “talk aloud.” These prompts asked learners to explain what they are seeing and thinking while watching the VR video, what they believe to be their priorities in managing care for the patient, what they find to be essential stimuli, and what they find to be distracting stimuli. After each VR viewing session, residents participated in a semistructured interview.

The 8 videos were also viewed by an expert physician within each discipline in surgery, emergency medicine, and anesthesia. These expert physicians also participated in “talk alouds” and reflected on aspects of the videos they believed learners might find challenging, stressful, distracting, and overwhelming. They also identified key learning objectives for junior residents in their respective specialties.

The residents and attending physician responses from their “talk alouds” and semistructured interviews were analyzed by the research team to identify thematic sources of cognitive load. We identified 5 sources: (1) acclimating to the chaos of the room and understanding the roles and priorities of the trauma team, (2) understanding and applying the protocol for evaluating and caring for a trauma patient per the ATLS guidelines, (3) anticipating next steps in care to manage the patient, (4) understanding clinical content, and (5) orienting to VR. Based on their content, 2 videos were then assigned to each of the first 4 categories. One video was created to orient the learner to experiencing the capabilities of cineVR.

Between 3 and 8 teaching points were created for each video and synchronized to the appropriate time in the video. The editing team created graphics using Adobe Photoshop software. The graphics were then animated using Adobe After Effects software before importing the animated graphics into the Adobe Premiere Pro editing software. For 2 of the videos, a script was written, and audio was recorded to be inserted as “voiceover.” The augmented videos were reviewed by the attending physicians, and further edits were made to improve timing and content.

### Lessons Learned

After going through the processes of preparation, set-up, filming, editing, and curriculum development in creating the trauma-training cineVR videos, our team has gathered a set of recommendations to guide other medical institutions interested in creating such a curriculum. Overall, our team learned the importance of working collaboratively and closely with both production partners and institutional partners, and testing equipment and filming scenarios beforehand allow the entire team and all stakeholders to be prepared and ready for filming day. Thinking through the logistics and workflows for each process, along with gaining appropriate approvals, can help streamline your filming and production and yield valuable cineVR videos. In Textbox 1, we summarize our recommendations for readers looking to embark on a similar process.

**Planning recommendations for each of the steps to create a 360-degree video and audio (cineVR) curriculum.**

Finding a production partner

- Conduct a broad search to identify a partner, including an online search, a literature search, and reaching out to virtual reality experts. Depending on your specific needs, your partner might be an industry partner or an academic center.
- Prioritize production partners with experience in health care.
- Ensure that they have the necessary technical infrastructure to maintain data security and Health Insurance Portability and Accountability Act (HIPAA) compliance.
- Seek partners who are open to collaboration.

Selecting recording perspectives

- Collaborate with a diverse cohort of physicians and staff to identify the perspectives that must be captured.

Equipment selection

- Evaluate the 360-degree video and audio recording technologies to optimize for audio and image quality, auto-stitching capability, size, and weight, as well as ease of use. Additional features to consider include external or internal battery and cost.

Setting up and filming the 360-degree videos

- Iteratively test different camera configurations in the room with providers and staff to ensure that the equipment does not get in the way of patient care. Run simulations of the patient care scenario to identify whether cameras have the potential to obstruct care or capture suboptimal video.
- Partner with the facilities team at your institution, as they can provide additional support, equipment, and advice to ensure that the equipment is tethered safely and within institutional guidelines.
- Avoid technical complications by having 2 sets of all filming equipment.

File management and video editing

- Create a standard file management protocol, followed by both the research and filming team.
- Appoint a data security lead that can communicate with the filming team and ensure file organization throughout the process.
- Blur any parts of the video that involve information or an individual that was not consented for (eg, an individual, not part of the filming, who walks past an open door).

Data protection and privacy

- Consult your institution’s data security, compliance, information technology, and legal teams early in the planning process to develop a data security and monitoring plan that adheres to all necessary guidelines.
- Develop a patient consent plan that tracks each patient to every part of their hospital stay.
- Co-develop a surrogate consent protocol with your institution’s institutional review board.
- Obtain consent from providers, staff, and other participants before filming, when possible. Attend change of shift and staff meetings to present the project, answer questions, and obtain consent.
- To capture any remaining providers, staff, police, emergency medical services, or any other individuals who might be filmed, station research staff outside each door of the trauma bay to ensure that all participants are consented.

Curriculum development

- Collaborate with educators, learners, and other stakeholders to iteratively design the curriculum.

## Discussion

This study demonstrates the feasibility of developing a cineVR-based training curriculum using real patient-care scenarios at an academic medical center. We illustrate important lessons learned from our experience in the hope other institutions may refer to this publication when planning similar projects. Important considerations include finding a production partner, choosing individual camera perspectives, setting up and filming the 360-degree videos, video editing and file management, data protection and privacy, and building the curriculum.

While this process requires significant time investment by medical and research personnel and extensive coordination across multiple stakeholders, these videos can be used for a variety of curricular needs. In the long-term, such video libraries, capturing a breadth of typical cases in trauma care, can be shared or exported to other trauma centers and teaching hospitals nationally.

The use of cineVR technology for medical simulation training is unique and promising because it addresses a major critique of traditional medical simulation: the lack of fidelity to real-life situations [10]. Currently, no simulation realistically depicts all of the physiological, mental, and behavioral components of patient care [27]. Poorly designed simulations can neglect important components of patient scenarios, suggesting that those missing components are unimportant or unintentionally encouraging shortcuts [28].

In studying simulation training in other disciplines, it has been shown that psychological fidelity is more important than physical fidelity [10]. The videos described in this article illustrate 2 separate but related psychological concepts in the virtual reality literature: “presence” and “PREality.” “Presence” is a person's subjective sensation of being in a (usually virtual) scene, enabling them to interact with and feel connected to the world outside their physical bodies via technology [29]. “PREality” is designed to prepare the viewer for an anticipated experience by placing them in the actual environment where they intend to work [30,31]. It forces a sense of déjà vu on the viewer, allowing them to connect more closely to a specific environment or activity. This is a different psychological phenomenon than the idea of presence. Although 360-degree videos do not have to be location-specific to create a sense of presence, they do need to be location-specific to create a sense of “PREality.” The methods to produce cineVR videos described here use both presence and PREality to establish a high level of psychological fidelity.

Until now, the extent to which cineVR has been used in medical education has been limited, with videos focused primarily in an operating room setting, using real or simulated patients [11,32]. Educational research on the potential of cineVR to augment trauma response training is extremely limited; to our knowledge, only one other study has been published [33]. In this study, nursing students viewed 360-degree video of trauma teams in Sweden using a desktop computer, and the analysis found that the tool can be a useful addition to existing methods in nursing education.

### Limitations

The video experiences produced at our institution may not be as applicable to learners at other institutions because of differing physical environments. Given the difference in environments, learners from other institutions will retain the sense of presence but will not obtain a sense of “PREality” when training through our curriculum. To combine presence and “PREality” with learning, institutions should develop their own training curriculum that is based in their particular environments.

### Next Steps

Our next steps are to test the feasibility, acceptability, and implementation of the curriculum described here in a proof-of-principle study among medical trainees. The specific details of the curriculum will be outlined in future publications. CineVR is a novel educational tool, and additional research can address several unanswered questions. For instance, there is limited research suggesting how to test the effectiveness of such a curriculum, how this technology can be used in team training, and the feasibility of incorporating additional capabilities such as gaze-tracking.

### Conclusion

It is feasible to develop a VR-based training curriculum using video from real patient-care settings. To do so, it is integral to partner with experienced organizations, plan and test filming processes, ensure robust data and physical security of all video and audio components, coordinate multistakeholder efforts, and be agile in unpredictable and uncharted environments. Although further research is required to understand the feasibility and effectiveness of such a curriculum, we hope the learning we gained from this process can guide other academic teaching hospitals and trauma centers who aim to develop high-fidelity training for their medical education programs.

## Appendix

Multimedia Appendix 1 Equipment specifications for the cameras and microphones.

## Abbreviations

ATLS: Advanced Trauma Life Support
cineVR: 360-degree video and audio
CLT: Cognitive Load Theory
ED: emergency department
EMS: emergency medical service
GRID: Game Research and Immersive Design
HIPAA: Health Insurance Portability and Accountability Act
IRB: institutional review board
VR: virtual reality
