# Supplementary material for: Developing Virtual Reality Trauma Training Experiences Using 360-Degree Video: Tutorial
Source: J Med Internet Res. 2020 Dec 16;22(12):e22420. doi: 10.2196/22420 (PMC7773512; doi:10.2196/22420)
Supplement: Multimedia Appendix 1 [file jmir_v22i12e22420_app1.docx]

**Appendix A**

Process for choosing cameras and microphones

The GRID Lab routinely tests the newest 360 cameras on the market. For this project, the had four key factors to consider when selecting the 360-degree camera: cost, image, weight and production efficiency. Production efficiency itself has three factors: whether the camera had auto-stitching capacity inside the camera prior to export, whether the camera used memory cards (or whether the camera had to be connected to a computer to export the footage) and whether or not the camera used removeable batteries (or did the camera itself have to be plugged into a power source to charge).

**Cost:**

From the outset, we knew that the project would require recording from five locations simultaneously. The efficiency of the Emergency Department demanded that cameras be swapped out immediately (rather than allowing time to download footage before replacing the camera in their positions. Therefore, two sets of cameras would be required, plus an additional two cameras in case of failure. This equation required a minimum of twelve cameras for the project. Budget limitations required us to only consider cameras below $750.00 (complete with batteries and memory cards). This requirement eliminated cameras such as the Go Pro Omni, which had a base cost of $5,000.00 at the time. Regardless of cost, it is important to note that most (of not all) professional and “pro-sumer” cameras such as the Go Pro Omni would have been eliminated anyway due to weight and logistical requirements (e.g.: the GPO weighs 2.32 pounds and has six camera – each requiring their battery and memory card to be replaced after every case; meaning that thirty batteries and thirty memory cards would have to be routinely replaced in a limited amount of time).

The cost factor reduced the GRID Lab considerations to seven of the newest 360-cameras on the market: (1) 360 Fly, (2) Bublecam, (3) Kodak PixPro, (4) LG 360, (5) Ricoh Theta S, (6) Samsung Gear 360 and (7) Yi 360. These seven cameras were field tested and well-researched to determine the optimal unit. Results of this study can be seen in the table (below); systematic reasoning for a stepped review follows.

**Weight:** Due to the five-pound suspended weight restriction from the state of California, we needed to keep the camera (with battery and memory card) to under two-thirds of a pound (10.56 oz). All seven cameras met this requirement.

**Efficiency:** Production efficiency was critical for this project, as the cameras had to be rotated back to the trauma bay as quickly as possible. Using two sets of cameras allowed for some flexibility, but we suspected that there may be instances where back-to-back cases might require a rotation in as quickly as fifteen minutes from the end of one case to the start of the next. For this reason, it was important for the camera to have removeable batteries and memory cards so that the cameras themselves were never taken “off-line”. The cameras were only unavailable for the time it took to replace these items (typically a matter of less than ten minutes for the entire set of cameras). Each camera had three batteries and two memory cards assigned to it.

Additionally, post-production efficiency was required as well. External stitching at the time required at least one hour (if not more) of computer and staff time per file (to upload, stitch, check and export). To complicate matters, most cameras only record four minutes per file. After four minutes of recording the camera starts a new file – which will need to be uploaded, stitched, checked and exported separately. In a scenario where we are recording 20-40 minute cases from five different camera angles, each case is producing between 25-50 files. If only twenty cases are recorded, the stitching alone would take one person two-to-five month to accomplish. Due to budget, internal stitching was a requirement.

Three cameras met the external battery requirement.

(2) Bublecam, (3) Kodak PixPro, and (7) Yi 360

Result: Four cameras met the external memory card requirement.

(2) Bublecam, (3) Kodak PixPro, (6) Samsung Gear 360 and (7) Yi 360

Result: Five cameras met the auto-stitching requirement.

(1) 360 Fly, (2) Bublecam, (5) Ricoh Theta S, (6) Samsung Gear 360 and (7) Yi 360

**Image:** 360-degree camera image can measure the quality of A) still images, B) video images that are auto-stitched, and C) video images stitched external to the camera. Our project was exclusively concerned with video images that are auto-stitched. Based on subjective evaluation, the GRID lab felt that 2800 pixel of horizontal resolution was the minimum amount required to have a quality experience.

Four cameras met the external memory card requirement.

(1) 360 Fly, (3) Kodak PixPro, (6) Samsung Gear 360 and (7) Yi 360

**Summary of Results:**

|  | 360 Video  Image Quality (stitched) | Weight | Production Efficiency | | |
| --- | --- | --- | --- | --- | --- |
|  |  |  | auto-stitching | external  memory card | external battery |
| 360 Fly | 2880 x 2880 | 6.06 oz | Yes | No: 32Gb internal | No |
| Bublecam | 1984 x 994 | 9.87 oz | Yes | Yes | Yes |
| Kodak PixPro | 5000 x 2500 | 9.03 oz | No | Yes (2) | Yes (2) |
| LG 360 | 2560 x 1440 | 2.65 oz | No | No: 4Gb internal | No |
| Ricoh Theta S | 1920 x 1080 | 4.40 oz | Yes | No: 8Gb internal | No |
| Samsung Gear 360 | 3840 x 1920 | 5.39 oz | Yes | Yes | No |
| Yi 360 | 3840 x 1920 | 5.99 oz | Yes | Yes | Yes |

At the end of the GRID Lab research, it was determined that four cameras met the image requirements for the project. In rank order of image quality, Kodak PixPro was the highest. Unfortunately, the camera did not have auto-stitching and actually required two external memory cards (one for each camera). This configuration would double the complexity of post-production. Therefore, the Pixpro was disregarded from consideration and three cameras were left in consideration: 360 Fly, Samsung Gear 360, and Yi 360.

The 360 Fly did not have external memory cards or an external battery, and therefore was eliminated from consideration. Final consideration was between two cameras: Samsung Gear 360 and Yi 360. Both cameras had identical image quality and similar weight. Both had auto-stitching and used external memory cards. The deciding factor was the use of an external battery. The Yi camera used external batteries while the Gear did not.

The GRID Lab did not compare microphones. At the time, immersive audio microphones below the $500 range were limited and the Zoom H2N (which met the weight requirement) was already in the GRID Lab inventory and known as a quality piece of equipment.
